# Brain-specific epigenetic markers of schizophrenia

**DOI:** 10.1038/tp.2015.177

**Published:** 2015-11-17

**Authors:** L F Wockner, C P Morris, E P Noble, B R Lawford, V L J Whitehall, R M Young, J Voisey

**Affiliations:** 1 Queensland Institute of Medical Research, Brisbane, QLD, Australia; 2 Department of Biomedical Sciences, Institute of Health and Biomedical Innovation, Queensland University of Technology, Brisbane, QLD, Australia; 3 Department of Psychiatry and Biobehavioral Sciences, University of California, Los Angeles, Los Angeles, CA, USA

## Abstract

Epigenetics plays a crucial role in schizophrenia susceptibility. In a previous study, we identified over 4500 differentially methylated sites in prefrontal cortex (PFC) samples from schizophrenia patients. We believe this was the first genome-wide methylation study performed on human brain tissue using the Illumina Infinium HumanMethylation450 Bead Chip. To understand the biological significance of these results, we sought to identify a smaller number of differentially methylated regions (DMRs) of more functional relevance compared with individual differentially methylated sites. Since our schizophrenia whole genome methylation study was performed, another study analysing two separate data sets of post-mortem tissue in the PFC from schizophrenia patients has been published. We analysed all three data sets using the *bumphunter* function found in the Bioconductor package minfi to identify regions that are consistently differentially methylated across distinct cohorts. We identified seven regions that are consistently differentially methylated in schizophrenia, despite considerable heterogeneity in the methylation profiles of patients with schizophrenia. The regions were near *CERS3*, *DPPA5*, *PRDM9, DDX43, REC8*, *LY6G5C* and a region on chromosome 10. Of particular interest is *PRDM9* which encodes a histone methyltransferase that is essential for meiotic recombination and is known to tag genes for epigenetic transcriptional activation. These seven DMRs are likely to be key epigenetic factors in the aetiology of schizophrenia and normal brain neurodevelopment.

## Introduction

Schizophrenia is a severe psychiatric disorder and affects 1% of the population. It has a high heritability of up to 80%^1, 2^ but despite decades of research, a large proportion of the genetic risk is still unaccounted for. A number of susceptibility genes located in neuronal signalling pathways such as dopamine and glutamate have been identified but the aetiology of schizophrenia remains largely unknown. Evidence now suggests that epigenetic modifications play a major role in schizophrenia pathophysiology.

DNA methylation reflects the addition of a methyl group on the cytosine of CpG dinucleotides. It is one form of epigenetic modification that regulates gene function, and this may be mediated through the environment.^3^ DNA methylation may alter gene expression by restricting access of transcription factors to promoter regions or by changing mRNA processing. Even though these cytosine modifications cannot be seen on the DNA sequence they can be inherited by offspring. In the past, schizophrenia DNA methylation studies were focused on peripheral tissue, mainly due to the limited access to post-mortem brain tissue. Our previous study was the first to assess DNA methylation in post-mortem brain tissue from schizophrenia patients using the Illumina Infinium HumanMethylation450 Bead Chip.^4^ In addition to assessing DNA methylation at CpG islands and promoters, the chip also measures CpG ‘shores' and ‘shelves' which are regions known to also have altered DNA methylation.^5^ After adjusting for age and post-mortem interval, 4641 probes corresponding to 2929 unique genes were differentially methylated. More than 100 of these genes overlap with a DNA methylation study of peripheral blood from schizophrenia patients.^6^ Important DNA methylation changes have been reported in the early stages of development and suggest that aberrant DNA methylation during development could be critical for the pathogenesis of schizophrenia.^7^ We found that probes in the regions of *DNMT1*, *NOS1* and *SOX10* were differentially methylated in schizophrenia^4^ and it is known these genes are also differentially methylated from foetal to neonatal life stage.^7^

Although methylation of single CpG sites have been shown to be related to phenotypes^8^ or gene expression,^9^ it is believed that most functionally important findings will be associated with the methylation of genomic regions.^10, 11^ Until recently there has been a paucity of algorithms that have identified differentially methylated regions (DMR) in a statistically robust manner. The *bumphunter* function found in the Bioconductor package minfi^12^ implements a method^13^ to identify and attach statistical uncertainty to DMRs in the Infinium HumanMethylation450 array. Clusters of probes, with the maximum gap between adjacent probes no more than a defined distance apart, are used to estimate a smoothed estimate of the level of methylation across the region. Unlike other DMR detection algorithms designed for the 450K array,^14, 15^ the *bumphunter* function determines candidate DMR based on a resampling procedure and adjusts for other covariates such as age and sex.^16^ In conjunction with genomic annotation, it is hypothesised that DMR will provide a more meaningful understanding of the impact of methylation on the development of specific phenotypes.^10^

Despite an increasing number of studies that have looked at methylation patterns in schizophrenia patients the results have been inconsistent. This is potentially due to the heterogeneity of schizophrenia or the different tissue samples investigated. Since our schizophrenia whole genome methylation study was performed, another study analysing two distinct sets of post-mortem tissue in the prefrontal cortex (PFC) from schizophrenia patients has been published.^17^ The analysis of this data in conjunction with our previously published data enables identification of those regions that are consistently differentially methylated across distinct cohorts. Identification of these regions may improve our understanding of the molecular mechanisms underlying schizophrenia.

## Materials and methods

### Samples

Frontal cortex post-mortem brain tissue from individuals with DSM-IV (Diagnostic and Statistical Manual of Mental Disorders, 4th edition) diagnosed schizophrenia (*n*=24) and controls (*n*=24) was provided by the Human Brain and Spinal Fluid Resource Centre (HBSFRC; courtesy of James Riehl). Participants gave written consent through the HBSFRC for their brain tissue to be used for research into etiopathogenesis of human disease. Each sample consisted of a coronal section (7-mm thick) that had been quick frozen and a section of frontal cortex was dissected from each frozen section sample weighing 0.4–1.0 g. Extraction of DNA was performed at the UCLA Clinical Microarray Core Laboratory using the Roche MagNa Pure Compact (Roche, San Francisco, CA, USA). Ethics approval for the project was obtained from the Human Research Ethics Committee of the Queensland University of Technology.

### Illumina infinium humanmethylation450 beadchip

DNA samples were sent to the Australian Genome Research Facility (AGRF) and stored at −20 ºC. Quality checking of the samples was performed by Nanodrop Spectrophotometer (Nanodrop, Wilmington, DE, USA) and resolution on a 0.8% agarose gel. Samples were bisulphite converted with Zymo EZ DNA Methylation kit (Zymo Research, Irvine, CA, USA). GenomeStudio v2011.1 (Illumina, San Diego, CA, USA) with methylation module 1.9.0 software (Illumina), with the default Illumina settings and Illumina HumanMethylation450_15017482_v.1.2 manifest files was used in the methylation analysis. The Infinium platform assays >485 000 CpG sites, encompassing 99% of RefSeq genes. It covers 96% of CpG islands with multiple sites in the island, the shores (within 2 kb from CpG islands) and the shelves (>2 kb from CpG islands). It also covers CpG sites outside of CpG islands and DNase hypersensitive sites as well as incorporating miRNA promoter regions. All the Illumina quality controls were found to be in order which included sample-independent controls, sample-dependent controls, staining controls, extension controls, target removal controls, hybridisation controls, bisulphite conversion I and II controls, specificity controls, non-polymorphic controls and negative controls.

### Data processing

For methylation analysis, IDAT files were loaded into the R (3.2.1)^18^ environment using the Bioconductor (http://www.bioconductor.org) minfi package 1.14.0.^12^ Quality control measures were assessed and no outlier samples were detected. One sample was removed from further analysis as age and post-mortem interval was not available. Data-driven sex values were recorded for all samples using the getSex function in the minfi package. In one instance it was ambiguous as to what the true gender was; hence this sample was excluded from further analysis.

The arrays were then background and control normalised using the minfi package; this is roughly equivalent to the normalisation procedure in Genome Studio (Illumina). Finally, technical differences between Infinium I and Infinium II probes were removed using Subset-quantile Within Array Normalisation (SWAN), developed by Maksimovic *et al.*^19^ and available in the minfi package. The methylation status for each probe was recorded as a beta value that ranged between 0 and 1, where values close to 1 represent high levels of methylation and where values close to 0 represent low levels of methylation.

A detection *P*-value was calculated for all probes on all arrays. A *P*-value>0.05 indicates that the data point is not significantly different from background measurements. A total of 13 764 probes were removed from the analysis as at least one sample had a detection *P*-value>0.05. We removed probes that had an annotated SNP (dbSNP134) at the single-base extension (position 0) or CpG site (positions 1–2) as it is possible that SNP differences in these locations may manifest as differential methylation on the 450k array. All probes on the X and Y chromosome were also removed. The data are referred to as the HBSFRC data set (GEO accession number GSE61107).

### Publicly available data

#### PFC data

The processed and normalised beta values for two data sets described in the study by Pidsley *et al.*^17^ were downloaded from the Gene Expression Omnibus (GEO); these were the London Brain Bank for Neurodegenerative Disorders (LBBND data set; GEO accession number GSE61431) and the Douglas Bell-Canada Brain Bank, Montreal (DBCBB data set; GEO accession number GSE61380). From these data sets, the methylation beta values from the PFC only were extracted. For the LBBND data set there were 20 schizophrenia cases and 23 controls (after the exclusion criteria as in the study by Pidsley *et al.*^17^ was applied); and for the DBCBB data set there were 18 schizophrenia cases and 15 controls. The downloaded data set was pre-processed and normalised according to the study by Pidsley *et al.*^17^ using the dasen function described in Pidsley *et al.*^20^ All probes on the X and Y chromosome were also removed. The main difference between the procedures is that we do not perform any between-array normalisation. Aryee *et al.*^12^ indicate that their version of stratified normalisation ‘is not recommended for cases where global changes are expected, such as in cancer–normal comparisons.' However, they do note that the function dasen does decrease the between-array variability. Ultimately, we were concerned that between-array normalisation would remove biological, rather than technical variability.

### Assessment of cell composition differences

The CETS package in R^21^ was used to estimate the proportion of neuronal cells in the samples. As the proportion of estimated neuronal cells was significantly different in the HBSFRC and LBBND data sets (Supplementary Table 1), the top 10 000 probes known to be epigenotype-specific marks for brain cells^21^ were removed from the analysis in all three data sets.

### Differentially methylated region detection

To assess DMRs between groups, we adapted the bump-hunting technique previously described^13^ to the 450k array. Probes were assigned to clusters so that two neighbouring probes in the same cluster are separated by at most 500 bp. For each probe, we estimated the difference in the average logit of the beta values (otherwise known as *M*-values; as recommended by Du *et al.*^22^) between cases and controls, adjusted for sex, age and post-mortem interval, when available, and smoothed these estimated differences using running medians. For all data, an empirical distribution was created by performing 1000 bootstrap samples and candidate regions were chosen based on whether the smoothed estimated differences were greater than the 95% quantile of the empirical distribution. This resulted in cut-offs of 0.097, 0.124 and 0.224 for the LBBND, DBCBB and HBSFRC data sets, respectively. The GenomicRanges package in R^23^ was used to assess whether any of the significant hyper-DMR or hypo-DMR overlapped by at least 2 bp in all three PFC data sets.

### Sensitivity analysis

A sensitivity analysis was additionally performed to assess the difference in significant DMRs using a 97.5% cut-off for all data sets. In addition, probes were assigned to clusters so that two neighbouring probes in the same cluster are separated by at most 1000 bp (instead of 500 bp) using a threshold of both 95 and 97.5%. Finally, to investigate the sensitivity to cell composition, the 10 000 probes known to be epigenotype-specific marks for brain cells were included in the analysis using both a maximum gap of 500 and 1000 bp gap between adjacent probes.

### Annotation of regions

Data were plotted using the Gviz package in R^24^ and the CpG Islands and known genes were downloaded for the USCS Browser. Regions were further annotated using the *matchGenes* function in the minfi package.

## Results

### Differentially methylated regions in the HBSFRC data set

In the HBSFRC data set, 6868 candidate DMR were identified (cut-off *M*-value 0.224) and of these, 1550 DMR had a *P*<0.05 where *P* is the nominated false discovery rate. When the *P*-values were adjusted such that the family-wise error rate was <0.1 three regions were differentially methylated (Table 1). *RNF39* was found to be significantly differentially hypomethylated in the DBCBB data set when controlling the FDR<0.05 (Δ*M*=−0.235; *P*=0.005); however, was not significantly differentially methylated in the LBBND data set (Δ*M*=−0.137; *P*=0.173), despite being chosen as a candidate region.

### Overlapping DMRs in publicly available data sets

Using the LBBND data set 4120 candidate DMR were identified (cut-off *M*-value 0.097) and of these 596 had a *P*<0.05. Using the DBCBB data set 2172 candidate DMR were identified (cut-off *M*-value 0.124) and of these 296 had *P*<0.05. Full annotated lists of candidate regions for each data set are provided in the Supplementary Material (Supplementary Tables 2–4).

**Figure 1:**
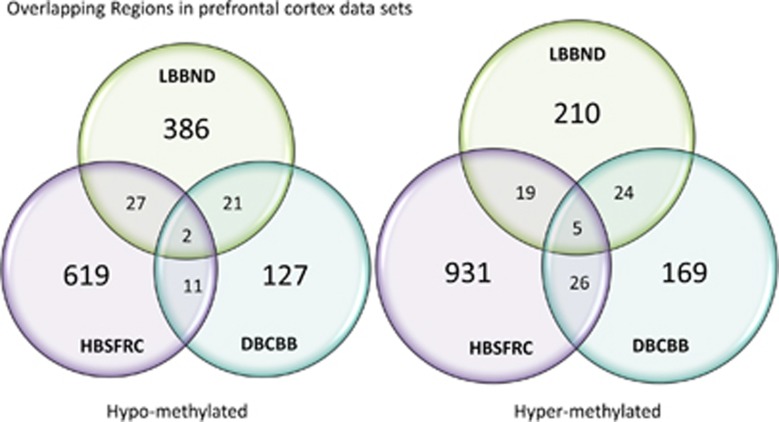
Summary of number of overlapping regions between the three prefrontal cortex data sets. DBCBB, Douglas Bell-Canada Brain Bank; HBSFRC, Human Brain and Spinal Fluid Resource Centre; LBBND, London Brain Bank for Neurodegenerative Disorder.

The significantly hyper- and hypomethylated regions were compared for overlaps. Figure 1 details the degree of the overlapping regions in the PFC data sets. Five regions were significantly hyper-methylated in all three PFC data sets (Table 2). Two regions were significantly hypomethylated in all three data sets (Table 2).

**Figure 2:**
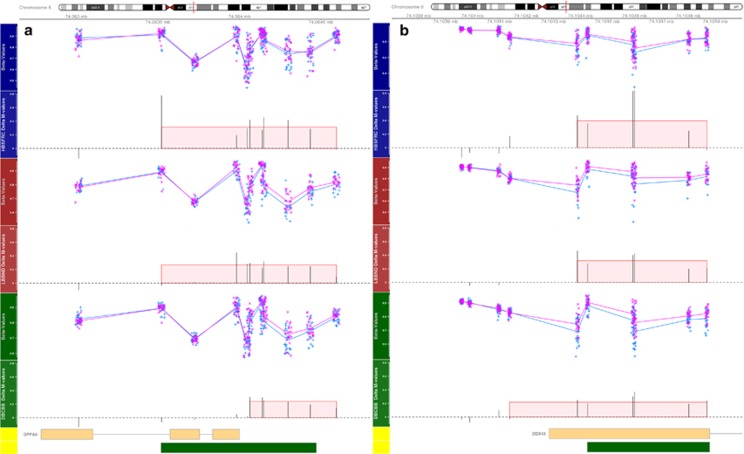
HBSFRC data set (blue axis), LBBND data set (red axis), DBCBB (green axis) with unadjusted beta values for the control (blue +) and schizophrenia groups (pink x) and the average difference in *M*-values for each probe; the width of the red highlighted area represents the identified differentially methylated regions, while the height of the region represents the average difference in *M*-values across the region. The yellow axis represents the known genes downloaded for UCSC browser and the green boxes represent known CpG islands: (**a**) region near *DPPA5*; (**b**) region near *DDX43*. DBCBB, Douglas Bell-Canada Brain Bank; HBSFRC, Human Brain and Spinal Fluid Resource Centre; LBBND, London Brain Bank for Neurodegenerative Disorder*.*

**Figure 3:**
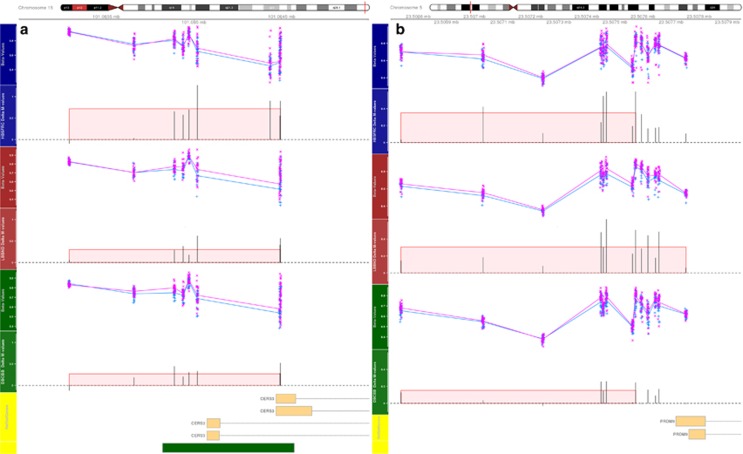
HBSFRC data set (blue axis), LBBND data set (red axis), DBCBB (green axis) with unadjusted beta values for the control (blue +) and schizophrenia groups (pink x) and the average difference in *M*-values for each probe; the width of the red highlighted area represents the identified differentially methylated regions, while the height of the region represents the average difference in *M*-values across the region. The yellow axis represents the known genes downloaded for UCSC browser and the green boxes represent known CpG islands: (**a**) region near *CERS3*; (**b**) region near *PRDM9*. DBCBB, Douglas Bell-Canada Brain Bank; HBSFRC, Human Brain and Spinal Fluid Resource Centre; LBBND, London Brain Bank for Neurodegenerative Disorder.

When these regions were annotated with known reference genes and CpG Islands from the UCSC browser, most regions were differentially methylated either upstream of, or on, the first exon of: Developmental Pluripotency Associated 5 (*DPPA5*), *DDX43* (Figures 2a and b); Ceramide Synthase 3 (*CERS3*), *PRDM9* (Figures 3a and b) and *REC8* (Supplementary Figure 1). One region was located on a CpG Island on chromosome 6 located in *LY6G5C* (Supplementary Figure 2) and another on chromosome 10, 131 kb upstream of the nearest gene *PFKP* (Supplementary Figure 3). More detailed annotation can be seen in Supplementary Table 5.

### Sensitivity analysis

When the maximum allowable gap between adjacent probes was changed from 500 to 1000 bp all seven regions remained significantly differentially methylated. Two additional regions near Piwi-Like RNA-Mediated Gene Silencing 1 (*PIWIL1*) and Succinate Dehydrogenase Complex, Subunit A, Flavoprotein Pseudogene 3 (*SDHAP3*) were also identified as being differentially methylated in all three data sets. When the *M*-value cut-off was defined to be greater than the 97.5% quantile of the empirical distribution, only the region near *CERS3* remained significant in all three data sets. However, *PRDM9*, *DDX43*, *LY6G5C* and the region on chromosome 10 had adjusted *P*-values<0.1 in all data sets. *DPPA5* was significant in two data sets (*P*<0.05) but had *P*=0.119 in the HBSFRC data set. *REC8* was not selected as a candidate region. Including the 10 000 probes known to be epigenotype-specific marks for neuronal cells made no discernible difference to the overlapping regions. Full details about overlapping candidate regions for all sensitivity analyses can be seen in Supplementary Table 6.

## Discussion

Using three publicly available data sets (including our study) that investigated tissue from the PFC of the brain, we have been able to identify seven regions that are consistently differentially methylated in schizophrenia. Six of these regions are located upstream or in exons of *CERS3*, *DPPA5*, *REC8*, *PRDM9, LY6G5C* and *DDX43*. One region on chromosome 10 is located more than 130 kb outside of the nearest gene *PFKP*. This region lies within a DNase hypersensitive site identified from the ENCODE project,^25^ suggesting it could be a marker of regulatory DNA.

The known characteristics of the three data sets presented here can be seen in Supplementary Table 7. All three data sets are not only geographically distinct but also cover a wide distribution of ages. The only substantial commonality is the diagnosis of schizophrenia. Thus, it can be expected that any differentially methylated overlapping regions represent the strongest evidence of schizophrenia-related methylation.

The DMRs associated with schizophrenia across all three brain data sets include biologically relevant regions that may explain some of the schizophrenia aetiology and represent genes important in neurodevelopment. *PRDM9* encodes a histone methyltransferase that specifically trimethylates H3K4 (Lys-4 in histone H3). Methylation of H3K4 is known to specifically ‘tag' genes for epigenetic transcriptional activation.^26, 27^ *PRDM9* is expressed in germ cells during meiotic prophase and its disruption inhibits prophase progression leading to sterility.^28^ Meiotic recombination events cluster into narrow segments of the genome, defined as hotspots and PRDM9 plays a role in determination of these recombination hotspots.^29, 30, 31^ In the past *PRDM9* was not on the radar as a schizophrenia candidate gene as genomic alterations that occur during meiosis are not detected using standard genetic mapping techniques. Future large scale studies that have access to pedigree information may uncover parent of origin effects.

The second DMR identified is located in the promoter region of a gene also important in meiosis. Meiotic recombination protein *REC8* encodes a member of the kleisin family of structural maintenance of chromosomes protein partners. *REC8* localises to the axial elements of chromosomes during meiosis in oocytes and spermatocytes.^32^ *REC8* was mapped to chromosomes 14q11.2-12,^32^ which is a potential schizophrenia susceptibility locus.^33^

*DDX43* encodes an ATP-dependent RNA helicase in the DEAD-box family. *DDX43* is implicated in sex-specific autosomal patterns^34^ and is thought to play a role in spermatogenesis.^35^ The region we identified in *DDX43* is also located within a CpG Island. *DPPA5* encodes an RNA-binding protein that is highly expressed in undifferentiated pluripotent stem cells and has an early embryo-specific expression pattern.^36^ The DMR we identified within *DPPA5* lies in a CpG Island. Another region that is differentially methylated in all three studies and lies within a CpG island is *CERS3. CERS3* contributes to the fatty acid composition and concentration of ceramides in the epidermis and male germ cells.^37, 38^ Although an association with schizophrenia has not been previously reported, ceramides are important in embryogenesis.^38, 39^ A deletion in this gene leads to autosomal recessive congenital skin disorder called ichthyosis.^40^ The DMR within *LY6G5C* also lies within a CpG island. Lymphocyte antigen-6 complex belongs to a cluster of leucocyte antigen-6 (LY6) genes located in the MHC class III region.^41^ MHC encodes 400 genes critical to immune system function and is strongly associated with schizophrenia susceptibility.^42^

A sensitivity analysis was also applied to the data allowing a 1000-bp gap between adjacent probes. All of the seven DMRs remained significant but two additional regions near *PIWIL1* and *SDHAP3* were also significantly differentially methylated in all three data sets. *SDHAP3* is a pseudogene and no information is available for the gene function. Interestingly, a region in this gene has been found to be differentially methylated in cerebellar tissue in an autism study.^43^ The autism study identified the DMR to be an important regulatory site from its location within a CCCTC factor-binding site.^43^ *PIWIL1* plays an important role in stem cell self-renewal, RNA silencing and translational regulation.^44^ More recently *PIWIL1* has been shown to play a crucial role in neuronal development.^45^ *PIWIL1* regulates polarisation and radical migration of newborn neurons in the developing cerebral cortex^45^ and copy number variations in this gene are associated with autism spectrum disorders.^46^

As a previous study has shown that the two major cell types, neurons and glia, have different DNA methylation signatures,^47^ we estimated the proportion of neuronal cells for each sample. Due to significant differences in cell composition in the HBSFRC and LBBND data sets between cases and controls, 10 000 probes known to be epigenotype-specific marks for neuronal cells^21^ were removed from all data sets. In addition, given that the proportion of neuronal cells was higher in the cases (compared with controls) in the LBBND data set, but lower in the cases (compared with controls) in the HBSFRC data set and approximately equal in the DBCBB data set, it is unlikely that the seven regions are consistently differentially methylated due to differences in cell composition.

Although different data normalisation techniques were used for the publically available data sets, the identification of seven significant DMRs points to the robustness of the results. Despite the considerable heterogeneity in the methylation profiles of patients with schizophrenia, consistent results were identified. The seven regions identified as differentially methylated from three independent sample sets could potentially establish a causal role in the pathology of schizophrenia-associated epigenetic modifications.

## Figures and Tables

**Table 1:** The average difference in *M*-values in those regions with a FWER <0.1 in HBSFRC data set

*Chr*	*Start*	*End*	*Average* ΔM	*FDR*	*FWER*	*Nearest gene*
chr13	100640914	100644657	0.624	1.8E−06	0.005	*ZIC2*
chr20	36148604	36149455	0.490	5.7E−06	0.015	*BLCAP*
chr6	30042137	30043924	−0.392	2.5E−05	0.065	*RNF39*

Abbreviations: FDR, false discovery rate; FWER, family-wise error rate; HBSFRC, Human Brain and Spinal Fluid Resource Centre.

**Table 2:** The average difference in *M*-values in regions consistently differentially methylated in all three prefrontal cortex data sets

*Chr*	*Average* ΔM *(HBSFRC)*	*Average* ΔM *(LBBND)*	*Average* ΔM *(DBCBB)*	*Nearest gene*
chr5	0.351	0.153	0.306	*PRDM9*
chr6	0.314	0.240	0.267	*DPPA5*
chr6	0.394	0.219	0.318	*DDX43*
chr6	−0.263	−0.156	−0.191	*LY6G5C*
chr10	0.365	0.286	0.241	*PFKP*
chr14	−0.368	−0.129	−0.150	*REC8*
chr15	0.715	0.269	0.301	*CERS3*

Abbreviations: DBCBB, Douglas Bell-Canada Brain Bank; HBSFRC, Human Brain and Spinal Fluid Resource Centre; LBBND, London Brain Bank for Neurodegenerative Disorders.
