# Supplementary figures and images for: Brain-specific epigenetic markers of schizophrenia
Source: Transl Psychiatry. 2015 Nov 17;5(11):e680–. doi: 10.1038/tp.2015.177 (PMC5068768; doi:10.1038/tp.2015.177)

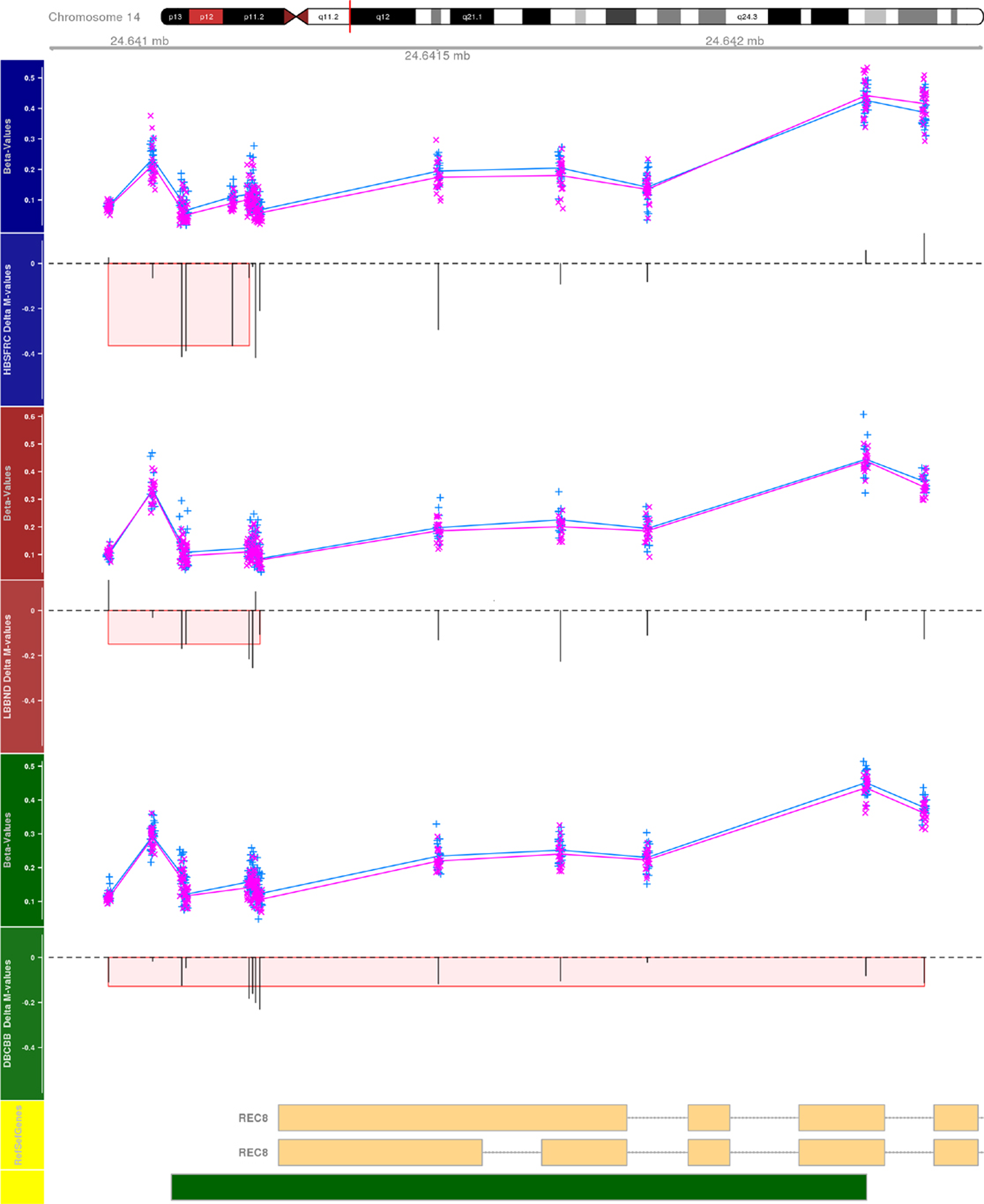

Supplement: Supplementary Figure 1 [file tp2015177x1.tif]

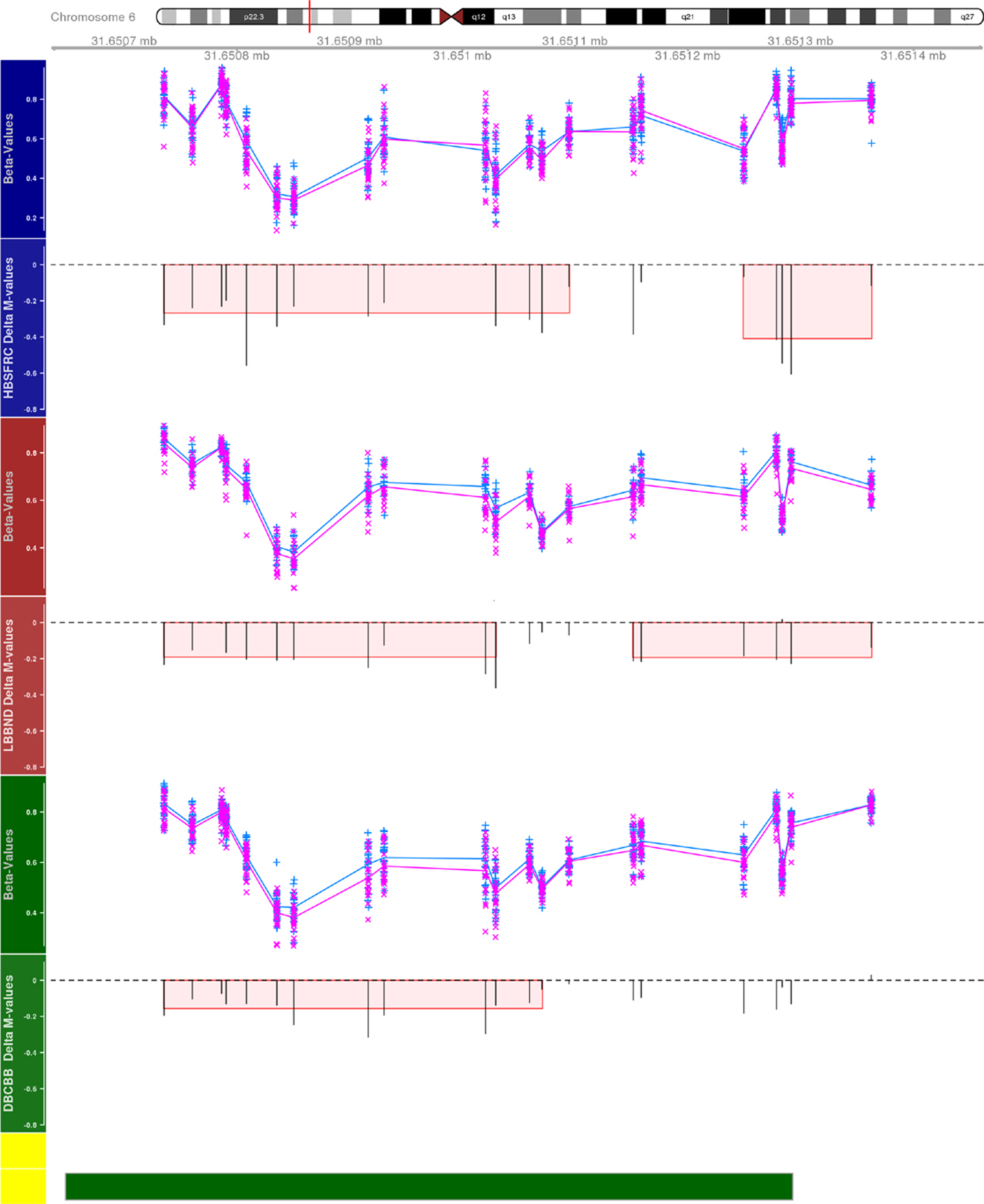

Supplement: Supplementary Figure 2 [file tp2015177x2.tif]

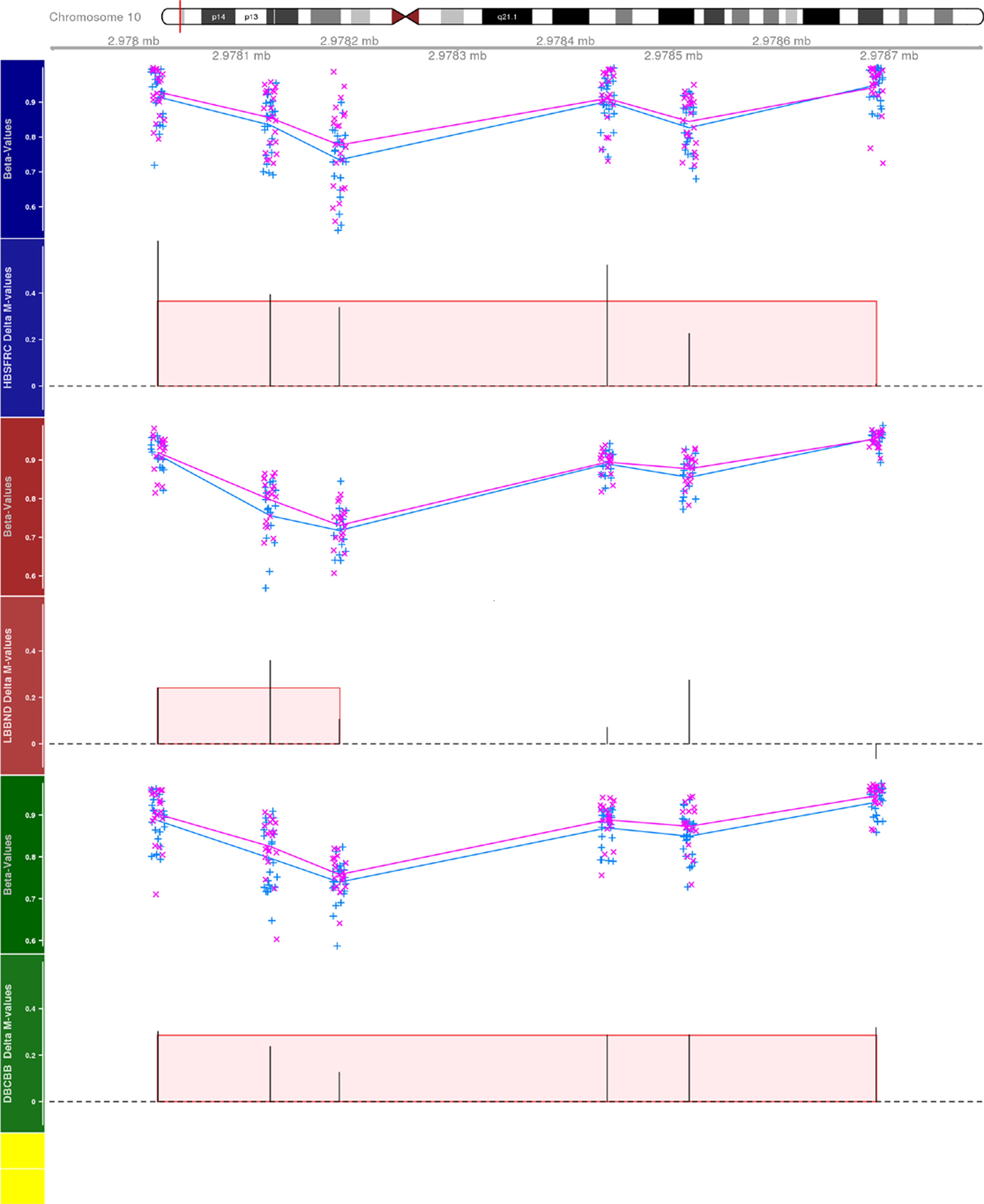

Supplement: Supplementary Figure 3 [file tp2015177x3.tif]
