# Supplementary material for: Brain-specific epigenetic markers of schizophrenia
Source: Transl Psychiatry. 2015 Nov 17;5(11):e680–. doi: 10.1038/tp.2015.177 (PMC5068768; doi:10.1038/tp.2015.177)
Supplement: Supplementary Table 1 [file tp2015177x4.doc]

**Supplementary Table 1:** Estimated proportion of neuronal cells.

|  |  | GSE61107 | | GSE61431 | | GSE61380 | |
| --- | --- | --- | --- | --- | --- | --- | --- |
|  |  | (HBSFRC) | | (LBBND) | | (DBCBB) | |
|  |  | **Control** | **Scz** | **Control** | **Scz** | **Control** | **Scz** |
| **Total** |  | 24 | 22 | 23 | 20 | 15 | 18 |
| Proportion of neuronal cells | mean | 0.362 | 0.235 | 0.402 | 0.463 | 0.383 | 0.414 |
|  | p-value | 0.002 | | 0.003 | | 0.193 | |
