# Supplementary material for: Brain-specific epigenetic markers of schizophrenia
Source: Transl Psychiatry. 2015 Nov 17;5(11):e680–. doi: 10.1038/tp.2015.177 (PMC5068768; doi:10.1038/tp.2015.177)
Supplement: Supplementary Table 6 [file tp2015177x9.doc]

**Supplementary Table 6:** Known characteristics of each data set

|  |  | GSE61107  (HBSFRC) | | GSE61431  (LBBND) | | GSE61380  (DBCBB) | |
| --- | --- | --- | --- | --- | --- | --- | --- |
|  |  | **Control** | **Scz** | **Control** | **Scz** | **Control** | **Scz** |
| **Total** |  | 24 | 22 | 23 | 20 | 15 | 18 |
| **Age** | mean | 71.3 | 51.3 | 62.0 | 62.1 | 42.2 | 45.5 |
|  | sd | 9.8 | 22.0 | 18.7 | 15.9 | 14.9 | 16.6 |
| **Male** | N (%) | 19 (79) | 15 (68) | 17 (74) | 11 (55) | 13 (87) | 15 (83) |
| **Female** | N (%) | 5 (21) | 7 (32) | 6 (26) | 9 (45) | 2 (13) | 3 (17) |
| **PMI** | mean | 14.1 | 24.0 |  |  |  |  |
|  | sd | 3.3 | 10.6 |  |  |  |  |
