# Supplementary material for: Brain-specific epigenetic markers of schizophrenia
Source: Transl Psychiatry. 2015 Nov 17;5(11):e680–. doi: 10.1038/tp.2015.177 (PMC5068768; doi:10.1038/tp.2015.177)
Supplement: Supplementary Legends [file tp2015177x11.doc]

**Supplementary Figure 1:** HBSFRC data set (blue axis), LBBND data set (red axis), DBCBB (green axis) with unadjusted beta values for the control (blue +) and schizophrenia groups (pink x) and the average difference in M-values for each probe; the width of the red highlighted area represents the identified differentially methylated regions, whilst the height of the region represents the average difference in M-values across the region. The yellow axis represents the known genes downloaded for UCSC browser and the green boxes represent known CpG islands: region near *REC8*

**Supplementary Figure 2:** HBSFRC data set (blue axis), LBBND data set (red axis), DBCBB (green axis) with unadjusted beta values for the control (blue +) and schizophrenia groups (pink x) and the average difference in M-values for each probe; the width of the red highlighted area represents the identified differentially methylated regions, whilst the height of the region represents the average difference in M-values across the region. The green boxes represent known CpG islands: region on chromosome 6 CpG island in *LY6G5C*

**Supplementary Figure 3:** HBSFRC data set (blue axis), LBBND data set (red axis), DBCBB (green axis) with unadjusted beta values for the control (blue +) and schizophrenia groups (pink x) and the average difference in M-values for each probe; the width of the red highlighted area represents the identified differentially methylated regions, whilst the height of the region represents the average difference in M-values across the region: region on chromosome 10.

**Supplementary Table 1:** Cell composition of data sets

**Supplementary Table 2-4:** Candidate regions for GSE61107 (HBSFRC); GSE61431 (LBBND); GSE61380 (DBCBB)

**Supplementary Table 5:** Annotation of the regions differentially methylated in all three data sets

**Supplementary Table 6:** Table of regions differentially methylated in the sensitivity analysis

**Supplementary Table 7:** Known characteristics of each data set
